# Effects of combined zinc and 2 mercaptobenzothiazole exposure on growth and physiological characteristics of *Lolium perenne* cultivars

**DOI:** 10.1039/d6ra04307k

**Published:** 2026-09-24

**Authors:** Yang Feng, Cheng Wu, Genshen Yin, Mingbiao Chen, Shah Fahad, Yanqun Zu, Zijin Hong, Gang Deng, Guofu Zhong

**Affiliations:** a College of Sanqi Medicine and Pharmacy, Wenshan University Wenshan 663099 China; b Faculty of Animal Science and Technology, Yunnan Agriculture University Kunming 650201 China zuyanqun@ynau.edu.cn; c College of Agriculture and Life Sciences, Kunming University Kunming 650214 China; d International College of Rivers and Ecological Securit, Yunnan University Kunming Yunnan 650500 China; e Department of Agronomy, Abdul Wali Khan University Mardan Khyber Pakhtunkhwa 23200 Pakistan shah_fahad80@yahoo.com; f State Key Laboratory of Vegetation Structure, Functions and Construction (VegLab), School of Agriculture, Yunnan University Kunming Yunnan 650504 China

## Abstract

Heavy metal and organic pollutant co-contamination is increasingly being detected in soils and water systems, particularly due to tire wear-derived compounds such as zinc (Zn) and 2-mercaptobenzothiazole (MBT), yet their combined effects on forage grasses remain poorly understood. This hydroponic study investigated the varied responses of 20 *Lolium perenne* cultivars to elevated zinc, 2-mercaptobenzothiazole, and their combined stress (Zn 20 mg L^−1^ (Zn treatment group), MBT20 mg L^−1^ (MBT treatment group), and MBT20 mg L^−1^ + Zn 20 mg L^−1^ (MBT–Zn treatment group)). Fifteen physiological and biochemical markers, including biomass, photosynthetic pigments, osmoregulatory chemicals, and antioxidant enzyme activity, were examined. The results demonstrated specific varietal adaptation mechanisms in response to both individual and combined stresses. Most varieties exhibited significant alterations in 13 of 15 traits compared to controls, except for underground biomass and soluble protein content. Across varieties, plant height, aerial/underground biomass, chlorophyll *a*/*b*, carotenoids, and root activity decreased under stress. In contrast, the levels of soluble protein, malondialdehyde (MDA), superoxide dismutase (SOD), and free proline exhibited significant increases. These changes were consistent with altered oxidative regulation and protective physiological responses under contaminant exposure. Combined Zn and MBT exposure produced trait-dependent and cultivar-dependent responses. Although combined exposure generally intensified oxidative responses, its effects on growth and photosynthetic traits were not consistently more severe than those of the individual stressors. Several cultivars showed partial attenuation of specific growth effects under combined exposure, indicating a nonadditive interaction between Zn and MBT rather than uniformly greater or lower toxicity. The findings provide a physiological basis for identifying *L. perenne* cultivars that maintain growth and protective metabolism under simultaneous Zn and MBT exposure. The identified tolerant cultivars represent candidates for further studies of contaminant uptake, translocation, and removal.

## Abstract 2

*Lolium perenne* exposed to Zn^2+^ metal stress, MBT organic stress and combined Zn^2+^ + MBT showed decreased plant height, shoot–root biomass and chlorophyll carotenoids with increased MDA, proline, SOD and CAT activities indicating antioxidant and osmotic adaptation responses.

## Graphical abstract

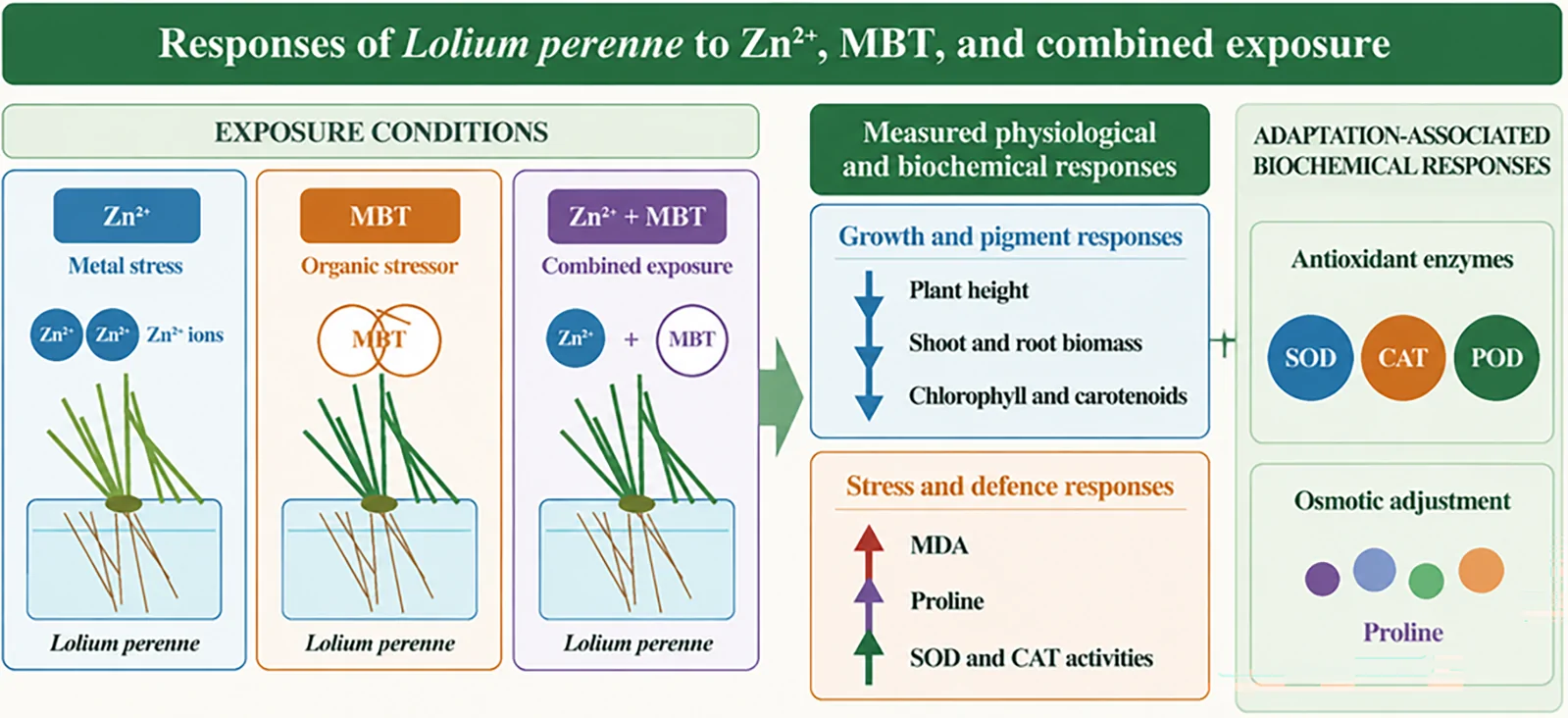

## 1 Introduction

Abiotic stress induced by pollution hinders plant growth by affecting biochemical, molecular, and physiological processes, leading to oxidative damage, membrane dysfunction, and reduced photosynthesis.^1^ Plants have evolved defense mechanisms, including the synthesis of metal-binding complexes like metallothioneins and phytochelatins, modification of root exudates to immobilize metals, and sequestration of metals within their vacuoles.^2^ Furthermore, beneficial microbes can aid in metal sequestration in soil and improve plant tolerance through the production of phytohormones and other compounds.^3^ Benzothiazole (BT) and its derivatives (BTs) are frequently utilized as vulcanization accelerators in rubber manufacturing,^4^ exhibiting extensive prevalence in environmental media and posing a considerable danger of malabsorption and bioaccumulation.^5^ Zinc (Zn), a standard constituent of rubber compounds that facilitates the vulcanization process, is found in tires at approximately 1–2% concentration.^6,7^ BTs and Zn as markers of tire wear particles are easy to leach into the soil or water environment with tire particles,^8^ thus causing plant growth inhibition.^9,10^ Zinc is an essential micronutrient that supports plant growth through roles in enzyme activity, protein structure, and antioxidant protection. Within the required range, Zn contributes to normal physiological function and does not act as a stress factor. However, excess Zn becomes phytotoxic, disrupts ion homeostasis and photosynthetic processes, and promotes oxidative stress. Therefore, in this study we treated Zn as a stressor only under elevated exposure conditions used to simulate contamination.^10^ In addition, the toxic effects of Zn at high levels on plants have been intensively studied.^11^

Plants exposed to both Zn and MBT stress demonstrate significant growth inhibition. This is seen in diminished shoot and root lengths, lower fresh and dry biomass, and general stunting. MBT can deteriorate in the environment, and its interaction with Zn seems to cause more significant physical harm, including disordered cellular structures, damaged organelles, and even cellular apoptosis, in contrast to exposure to a single stressor. Root development is notably susceptible, with substantial inhibition of both primary and lateral root elongation.^12^ The combined stress significantly affects the photosynthetic machinery. Plants exhibit a notable decline in net photosynthesis, transpiration, and stomatal conductivity.^12^ The levels of chlorophyll and carotenoids significantly decline, presumably because excess Zn displaces vital ions such as magnesium, hence hindering pigment production and functionality.^13^ The combined action of Zn and MBT markedly elevates oxidative stress, concentrations of reactive oxygen species, including superoxide anions and hydrogen peroxide, electrolyte leakage and malondialdehyde.^14^

*L. perenne*, often known as perennial ryegrass, is of considerable significance in China owing to its varied applications in agriculture and environmental management.^15^ It is primarily a crucial forage grass, extensively grown in pastures to supply high-quality, nutritious feed for animals, necessary for the ruminant farming sector.^16^ In addition to its application in animal husbandry, perennial ryegrass is widely utilized for establishing robust turf for lawns, recreational spaces, and sports fields, owing to its quick germination, powerful development, and capacity to develop a dense, resilient sward.^17^ Ecologically, its robust and wide fibrous root structure is highly efficient for soil conservation, aiding in the stabilization of soil and the prevention of erosion on slopes, riverbanks, and degraded terrains.^18^ This resilient vitality and strong characteristics render it a viable option for phytoremediation, capable of thriving in and aiding the restoration of soils contaminated with heavy metals such as copper, chromium, and lead.^19^

Although zinc is an essential micronutrient, elevated concentrations lead to phytotoxicity and oxidative stress; therefore, Zn is considered a heavy metal stressor under contaminated conditions. This study aimed to determine how individual and simultaneous exposure to Zn and MBT affects the growth, photosynthetic performance, oxidative status, and physiological regulation of *L. perenne*, and whether these responses vary among cultivars. We hypothesized that simultaneous Zn and MBT exposure would produce nonadditive physiological and growth responses that differ from those induced by either stressor alone. We further hypothesized that the direction and magnitude of the combined response would vary among traits and cultivars because of differences in antioxidant defence, osmotic adjustment, and overall stress adaptation capacity. This study evaluates the individual and interactive effects of Zn and MBT on multiple *L. perenne* cultivars. Combined contaminants do not necessarily produce a simple additive increase in toxicity because their interaction may be synergistic, additive, or antagonistic depending on pollutant chemistry, exposure conditions, plant genotype, and the physiological endpoint examined. Evaluating multiple growth, photosynthetic, and biochemical traits therefore provides an opportunity to determine whether concurrent Zn and MBT exposure produce a consistent toxic response or a more complex cultivar-dependent interaction. Although physiological responses to combined contaminants have been examined in several plant species, information on cultivar-dependent responses of perennial ryegrass to simultaneous Zn and MBT exposure remains limited. The novelty of the present study lies in the comparative evaluation of 20 *L. perenne* cultivars exposed to Zn, MBT, and their combination within a common experimental framework. By integrating growth, photosynthetic pigment, root activity, osmotic adjustment, and measured oxidative response traits, the study identifies cultivar dependent and trait dependent variation in responses to individual and combined contaminant exposure. This comparative approach further reveals that simultaneous Zn and MBT exposure produces complex and nonadditive responses rather than a uniform increase in toxicity, providing a physiological basis for identifying contrasting cultivars for further mechanistic and contaminant uptake studies.

## 2 Materials and methods

### 2.1 Experimental materials and reagents

The experimental materials are presented in Table 1, comprising 20 perennial types of *L. perenne*, and the 2-mercapto-benzothiazole (C_7_H_5_NS_2_) utilized in the experiment was acquired from Shanghai Yi En Chemical Technology Co., Ltd. Zn sulfate heptahydrate (ZnSO_4_·7H_2_O), dimethyl sulfoxide (DMSO), and copper sulfate pentahydrate (CuSO_4_·5H_2_O) are high-purity analytical reagents manufactured by Sinopod Chemical Reagent Co., Ltd. Dimethyl sulfoxide was used as a cosolvent to dissolve 2-mercaptobenzothiazole, and its final concentration was maintained at 0.1 percent in all relevant treatments. A solvent control containing 0.1 percent DMSO without Zn or MBT was included to exclude solvent effects. Copper sulfate pentahydrate was included only as a micronutrient component of the Hoagland nutrient solution and was not used as a stress factor. Soluble protein kits, proline kits, root activity kits, MDA kits, and antioxidant enzyme SOD kits were acquired from Beijing Solaibao Technology Co., Ltd. Pure water was produced by the laboratory's purification system.

**Table 1:** Names, origin and codes of 20 *Lolium perenne* varieties

Number	Name	Native	Code	Number	Name	Native	Code
1	Turfgold	Danish	JP	11	Yatsyn	USA	YQ
2	Flash green	USA	SD	12	New Sealand	United States	NSL
3	Turfsun	Danish	LT	13	Ray Jie	Germany	RJ
4	Neruda	Danish	MS	14	Ray hu	Germany	RH
5	Fairy green	America	ZL	15	Ray Li	Germany	RL
6	Royal green	USA	TL	16	*Lolium perenne*	NZ	MO
7	Mathilde	Denmark	MD	17	Bomber	America	HZJ
8	Nui	New Zealand	NY	18	Nebula	Argentina	XY
9	Quijote	Argentina	TJKD	19	Dina	Denmark	TN
10	Monro	Danish	ML	20	Lark	United States	BLN

### 2.2 Test methods

*L. perenne* seeds of equal size and full grain size were selected and disinfected with 10% H_2_O_2_ for 10 min, then washed several times with distilled water and dried for use. According to Habibul *et al.* (2019),^20^ an autoclave was used with deionized water, Hoagland solution, and culture bottles to prevent possible influence of microorganisms on the experiment, we autoclaved the culture bottles and the deionized water used to prepare the nutrient solutions. We also autoclaved the prepared Hoagland nutrient solution and conducted subsequent handling under sterile conditions to reduce microbial growth during the exposure period. After repeatedly washing the *L. perenne* seeds with ultra-pure water, the seeds were soaked in warm water for 2 h (surface sterilized the seeds with 10% H_2_O_2_ for 10 min and then rinsed them three to five times with sterile deionized water to remove residual disinfectant before germination), and germinated on sterile wet filter paper in a growth chamber with a diameter of 9 cm in a culture dish at 25 °C. All culture bottles were placed in a controlled growth chamber under the following conditions: temperature 25 degrees Celsius, 16-hour light and 8-hour dark photoperiod, and light intensity of 2100 to 2500 Lux.

#### 2.2.1 Experimental design, replication, and randomization

The experiment followed a completely randomized factorial design comprising 20 *L. perenne* cultivars and four exposure treatments, namely control, 20 mg per L Zn, 20 mg per L MBT, and 20 mg per L Zn plus 20 mg per L MBT. Three independently prepared culture bottles were established for each cultivar and treatment combination. Each culture bottle contained ten uniform seedlings and constituted one independent experimental unit and one biological replicate. Thus, the complete experiment comprised 240 independent culture bottles. Seedlings within the same culture bottle shared the same nutrient solution and treatment environment and were therefore considered subsamples of the culture bottle rather than independent biological replicates. Technical replicates, where performed, represented repeated analytical measurements of material derived from the same biological replicate and were not considered independent experimental units. The nutrient solutions were renewed every three days.

The concentrations of Zn and MBT were each set at 20 mg L^−1^ as controlled high exposure challenge levels. These concentrations were selected on the basis of preliminary range-finding experiments in which they produced consistent and measurable changes in the growth and physiological characteristics of *L. perenne* without causing complete seedling mortality during the 14-day exposure period. The purpose of using equal nominal concentrations was to facilitate comparison of the individual and combined treatments and to distinguish cultivar-specific stress responses. These concentrations were not intended to represent the levels normally detected in surface water, roadway runoff, or roadside soil. Reported environmental concentrations are generally lower. Unfiltered Zn concentrations in highway runoff have been reported at approximately 0.047 to 3.06 mg L^−1^, depending on pavement type and runoff event. Total benzothiazole concentrations in municipal wastewater effluents were reported at 1.9 to 6.7 µg L^−1^, while street runoff contained concentrations approximately one order of magnitude higher. Benzothiazoles have also been detected in tire wear particles and road dust, although concentrations expressed per unit mass of solid material cannot be directly compared with dissolved concentrations in hydroponic solution. Therefore, the present treatment represents a high exposure mechanistic scenario used to evaluate comparative plant responses rather than a direct simulation of typical environmental contamination.

The four exposure treatments were the control, 20 mg per L Zn, 20 mg per L MBT, and 20 mg per L Zn plus 20 mg per L MBT. Dimethyl sulfoxide was used to dissolve MBT, and its final concentration was maintained at 0.1% in the MBT-containing treatments. A corresponding solvent control containing 0.1% DMSO without Zn or MBT was included. The concentration settings of ZnSO_4_·7H_2_O determined by literature reports and pre-experiments were as follows: control group 0 mg L^−1^ (CK), Zn 20 mg L^−1^ (Zn treatment group), MBT20 mg L^−1^ (MBT treatment group), and MBT20 mg L^−1^ + Zn 20 mg L^−1^ (MBT–Zn treatment group). 20 mg L^−1^ as the exposure concentration for both Zn and MBT. According to the pre-experiment, 0.1% (volume fraction) DMSO was added as a cosolvent. The control group was each was divided into two groups: the polluted liquid was a 0.1% (volume fraction) DMSO solution (control), and ultra-pure water (blank control). Zn was added as ZnSO_4_·7HO_2_. The culture medium was changed every three days for the experimental group. After 14 days of exposure, the plants were harvested, divided into roots and leaves, weighed, and stored in a sealed sample bag at −20 °C until extraction.

### 2.3 Determination parameters

#### 2.3.1 Determination of morphological parameters

Assessments were conducted 14 days post-transplantation, and five plants from each group were chosen to harvest to statistically document the number of roots per plant, as well as measures of plant and root length. Five plants from each group were picked at random. The height and root length of *L. perenne* were measured using a vernier caliper. Assessment of fresh weight (FW): the aerial and root components of *L. perenne* were segregated, washed with deionized water, and weighed following surface drying with a hygroscopic agent to ascertain the fresh weight of both the aerial and subterranean sections. The green grass was dehydrated at 105 °C for 2 hours, thereafter baked to a constant mass at 60 °C, and its dry mass was measured.

#### 2.3.2 Determinations of physiological parameters

For the determination of the photosynthetic pigment content, fresh *L. perenne* leaves weighing approximately 0.1 g were cut up and placed in a test tube. Add 10 mL of 95% ethanol and stand 24 h away from light until the green leaf tissue becomes colorless. 95% ethanol was used as a blank control. The light absorption values and photosynthetic pigment contents of chlorophyll *a* (Chl *a*), chlorophyll *b* (Chl *b*), total chlorophyll (Chl), and carotenoid (C *x*·*c*) were determined by a spectrophotometer at 470 nm, 645 nm, 649 nm, and 663 nm, respectively, in mg per g FW.^49^ These physiological indicators are calculated as follows:

1 Chl *a* = (13.95 × *A*_665_ − 6.88 × *A*_649_) × *V* × *N*/*W*

2 Chl *b* = (24.96 × *A*_649_ − 7.32 × *A*_665_) × *V* × *N*/*W*

3 Total Chl = Chl *a* + Chl *b*

4 Carotenoid = (1000 *A*_470_ − 2.05 Chl *a* − 114.8 Chl *b*)/245 × *V* × *N*/*W*

where *V* is the extraction liquid volume (mL), *N* is the dilution ratio, and *W* is the fresh weight (g) of the sample.

Weigh 0.1 g *L. perenne* tissue in the research body, add a modest amount of quartz sand and 2 mL 10% trichloroacetic acid, and grind it into a homogenate. The homogenate is centrifuged at 4000 g for 10 min, and the supernatant is the crude enzyme liquid, which is stored in the refrigerator at 4 °C to be measured. Soluble protein content, proline content, root activity, MDA content, and antioxidant enzyme SOD activity of *L. perenne* were determined according to the methodology suggested by ref. 50.

### 2.4 Data collation and analysis

Microsoft Excel 2021 software was utilized for data sorting, while SPSS 27.0 software was employed for variance analysis, factor analysis, correlation analysis, and principal component analysis. GraphPad 10 software was used for plotting. Data were expressed as mean ± SD (standard deviation). One-way ANOVA was conducted for ANOVA, and DUNCAN's method was applied for multiple comparisons in case of significant or highly significant differences. The membership function method was adopted to evaluate the difference in Zn and MBT tolerance of perennial *L. perenne* seedlings by calculating each value through the ratio of the data of each trait under stress treatment group and normal control (CK) treatment.^21^

Membership function values were calculated using:

5 *µ*(*X*_*i*_) = (*X*_*i*_ − *X*_min_)/(*X*_max_ − *X*_min_), *i* = 1, 2, …, *n*

where, *X*_*i*_ represents *i* comprehensive index; *X*_min_ is the minimum value of *i* comprehensive index; and *X*_max_ is its maximum value.

The comprehensive index weights are calculated as follows:

6 *w*_*i*_ = *p*_*i*_/∑*p*_*i*_, *i* = 1, 2, …, *n*

where, *w*_*i*_ denotes the weight of *i* comprehensive indicator among all comprehensive indicators; *p*_*i*_ refers to the contribution rate of each variety's *i* comprehensive indicator.

The calculation method of comprehensive valuation is as follows:

7 *D* = ∑[*µ*(*X*_*i*_) × *w*_*i*_], *i* = 1, 2, …, *n*

*D* represents a measure of tolerance level evaluated by considering various indices under stress conditions.

## 3 Results

### 3.1 Analysis of variation of index traits of *L. perenne* varieties

The results of 15 traits of *L. perenne* showed (Table 2) that under single and combined Zn and MBT stress, compared with the control group, except for underground biomass and soluble protein content, changes of the remaining 13 traits were significantly different. The number of tillers and some physiological indexes (SOD activity, proline and MDA content) increased compared with the control group, but the other indexes decreased. In the control group, coefficient of variation for all traits ranged from 17.16% to 44.45%.

**Table 2:** Variation statistics of relative indices of seedling traits of *Lolium perenne* under Zn and MBT stress^a^

Morphology	Control group		Zn treatment group			MBT treated group			MBT–Zn treatment group		
	Mean ± standard deviation	Coefficient of variation (%)	Mean ± standard deviation	Coefficient of variation (%)	Rate of change (%)	Mean ± standard deviation	Coefficient of variation (%)	Rate of change (%)	Mean ± standard deviation	Coefficient of variation (%)	Rate of change (%)
Above ground biomass (mg FW)	53.82 ± 21.11^a^	39.22	42.78 ± 20.58^b^	48.12	−20.51	43.94 ± 19.60^b^	44.60	−18.34	42.88 ± 17.92^b^	41.79	−20.32
Underground biomass (mg FW)	13.33 ± 5.93^a^	44.45	12.25 ± 5.62^a^	45.91	−8.11	12.52 ± 6.08^a^	48.56	−6.06	11.37 ± 5.39^a^	47.38	−14.69
Dry weight (mg DW)	8.16 ± 2.83^a^	34.71	6.34 ± 2.62^b^	41.32	−22.31	6.32 ± 2.39^b^	37.77	−22.56	6.19 ± 2.36^b^	38.07	−24.23
Plant height (cm)	18.68 ± 4.14^a^	22.15	16.73 ± 3.59^b^	21.49	−10.49	17.33 ± 3.78^ab^	21.82	−7.26	16.23 ± 4.61^b^	28.38	−13.15
Root length (cm)	8.32 ± 2.38^a^	28.60	6.98 ± 2.27^b^	32.59	−16.12	6.05 ± 2.26^c^	37.34	−27.27	6.16 ± 1.35^c^	22.00	−25.99
Number of tillers	5.72 ± 1.60^a^	27.91	5.73 ± 2.01^a^	35.01	0.29	6.38 ± 1.97^a^	30.81	11.66	6.28 ± 2.32^a^	36.96	9.91
Chlorophyll *a* (mg per kg FW)	0.82 ± 0.14^a^	17.54	0.61 ± 0.13^b^	22.24	−25.83	0.64 ± 0.14^b^	22.06	−21.63	0.66 ± 0.13^b^	19.70	−19.62
Chlorophyll *b* (mg per kg FW)	0.22 ± 0.04^a^	18.10	0.19 ± 0.05^b^	26.31	−15.83	0.18 ± 0.04^b^	21.93	−17.56	0.19 ± 0.05^b^	23.66	−14.78
Carotenoids (mg per kg FW)	0.18 ± 0.03^a^	18.49	0.13 ± 0.035^b^	26.96	−28.49	0.14 ± 0.03^b^	21.24	−21.08	0.15 ± 0.03^b^	18.69	−19.21
Total chlorophyll (mg per kg FW)	1.04 ± 0.18^a^	17.16	0.79 ± 0.18^b^	22.81	−23.65	0.82 ± 0.18^b^	21.74	−20.74	0.84 ± 0.16^b^	19.24	−18.83
SOD activity (U per g mass)	209.06 ± 70.51^b^	33.73	250.51 ± 70.16^a^	28.01	19.82	249.67 ± 76.72^a^	30.73	19.42	253.20 ± 95.29^a^	37.63	21.11
Proline content (µg per g FW)	31.80 ± 9.55^b^	30.02	37.61 ± 9.97^b^	26.51	18.27	38.70 ± 10.38^a^	26.82	21.72	39.11 ± 11.00^b^	28.13	23.02
MDA content (nmol g^−1^)	9.09 ± 1.82^c^	19.97	10.40 ± 2.68^b^	25.79	14.48	10.96 ± 2.74^b^	24.97	20.66	12.01 ± 2.86^a^	23.80	32.11
Soluble protein content (µg g^−1^)	38.94 ± 9.18^a^	23.58	35.35 ± 10.73^a^	30.35	−9.22	37.25 ± 10.18^a^	27.33	−4.35	39.09 ± 11.66^a^	29.83	0.39
Root activity	353.42 ± 98.30^a^	27.81	288.58 ± 65.89^b^	22.83	−18.35	287.29 ± 71.63^b^	24.93	−18.71	278.79 ± 75.69^b^	27.15	−21.12

a Different lowercase letters after the same row of data indicate a significant difference in an index between Zn and MBT treatments (*P* < 0.05).

The index exhibiting the lowest coefficient of variation is total chlorophyll, while the greatest is underground biomass. In the Zn treatment group, the coefficient of variation for characteristics varied from 21.49% to 48.12%, with plant height exhibiting the lowest coefficient and above-ground biomass the highest. In the MBT treatment group, the coefficient of variation for characteristics varied from 21.24% to 48.56%, with carotenoid content exhibiting the lowest coefficient and underground biomass the highest. In the MBT–Zn treatment group, the coefficient of variation in traits ranged from 18.69% to 47.38%, with the smallest coefficient of variation index being carotenoid content and the largest being underground biomass. This study shows that there is a rich variety of *L. perenne*-related traits under different treatment conditions, with large differences between traits. A single index is not able to fully assess the tolerance of different grass varieties.

### 3.2 Effects of combine Zn and 2 mercapto-benzothiazole stress on *L. perenne* seedling growth

As can be seen in Fig. 1A, the above-ground biomass of *L. perenne* under single and combined Zn and MBT stresses decreases to varying degrees compared to the control. Above-ground biomass of Fairy Green under MBT stress decreased the most by 53.09%, while that of Ray under Zn and MBT–Zn stress decreased the most by 63.12% and 49.47%, respectively. In the treatment group, above-ground biomass of New Sealand, Flash Green, and Fairy Green exhibited an increase under combined stress of Zn and MBT treatments, in contrast to the individual stresses of Zn and MBT. Fig. 1B illustrates that subsurface biomass trends of *L. perenne* vary among the different types in comparison to the control group. The underground biomass of Mathilde, Turfsun, Nui, Quijote, Monro, Yatsyn, and Mathilde rose by 134.68%, 131.25%, 104.55%, 107.35%, and 101.33%, respectively, with no significant differences observed. The underground biomass of alternative *L. perenne* variants diminished greatly.

**Fig. 1:**
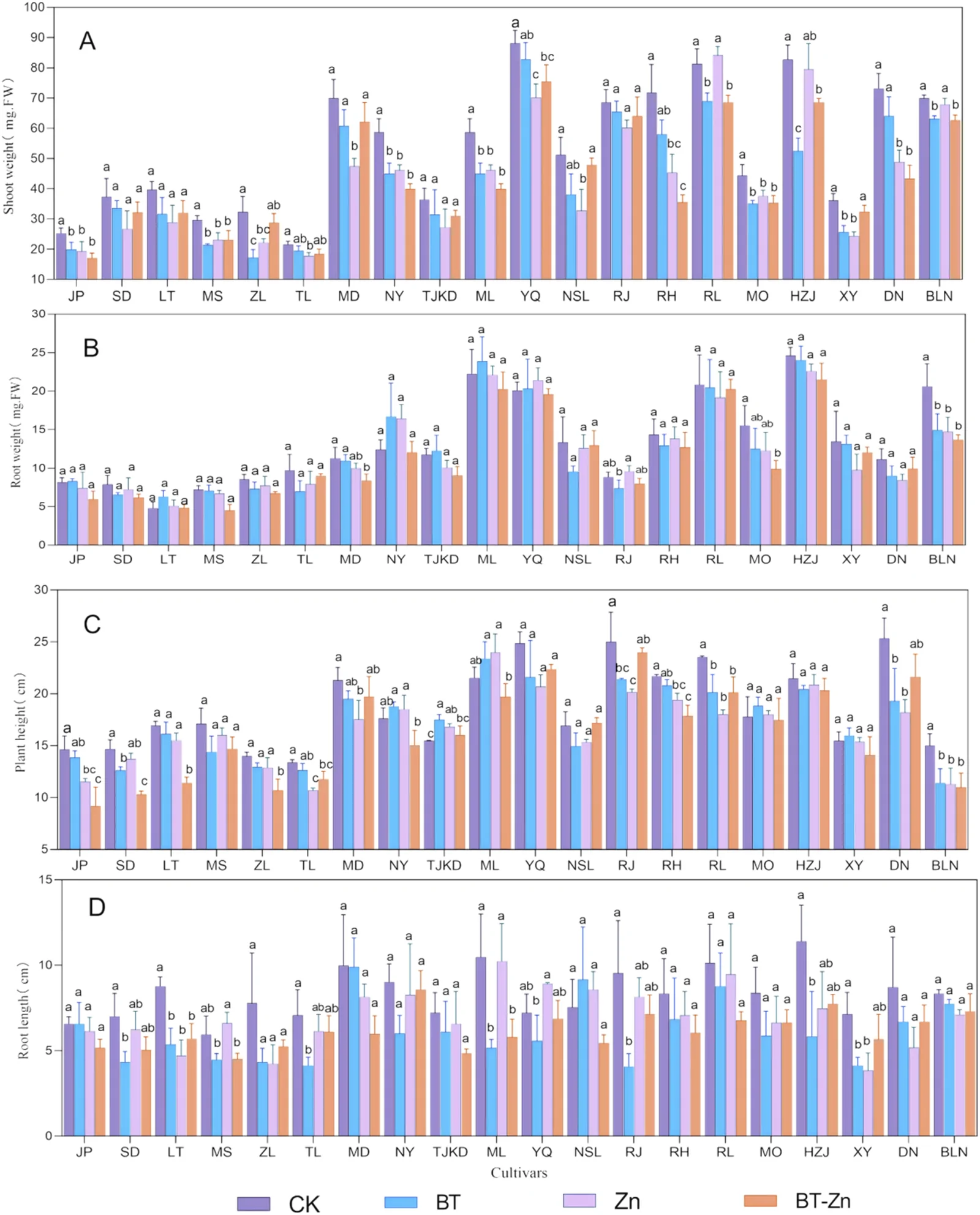
Effects of zinc and 2 mercaptobenzothiazole stress on aboveground biomass (A), underground biomass (B), plant height (C) and root length (D) of *Lolium perenne*. Different lowercase letters indicated that the same *Lolium perenne* had significant differences among different stress concentrations (*P* < 0.05).

As can be seen in Fig. 1C, the variation trends of plant height are different for the different varieties, with all treatment groups showing a decreasing trend compared to the control group except for the Quijote and Monro varieties. Under the single and combined effects of Zn and MBT, the largest increases were found in Quijote (113.15%), Neruda (111.11%) and Quijote (103.45%), respectively. Lark had the largest decrease in plant height under MBT treatment (76.00%), Turfgold had the largest decrease under Zn and MBT–Zn stress (83.17% and 62.64% in control group, respectively). As can be seen in Fig. 1D, the root lengths of all the varieties except New Sealand, which increased by 121.68% and 113.72% under treatment with Zn and MBT respectively, showed a decreasing trend compared to control under different treatment conditions. Under combined stress of Zn and MBT treatments, root lengths of the fairies and nebulae increase compared to the case of Zn and MBT stress alone.

As shown in Fig. 2A, the dry weight of different *L. perenne* species decreased to different degrees compared to the control group. Except Turfsun, Royal Green and Quijote, the dry weight of other *L. perenne* varieties decreased significantly. Above-ground biomass decreased most in the MBT treated group with Nebula (58.82%) and in the Zn and MBT–Zn treated groups with Fairy Green and Turfgold (59.12% and 56.55%, respectively). Fig. 1B illustrates that the trend of tiling number index diverges from that of the other growth indicators. The patterns in tiling numbers for various breeds markedly differed from those of the control group. The underground biomass of the Quijote, Ray hu, Bomber, and Nebula types exhibited a statistically significant difference from the control group; however, variation in tillering number among other *L. perenne* varieties was not significant.

**Fig. 2:**
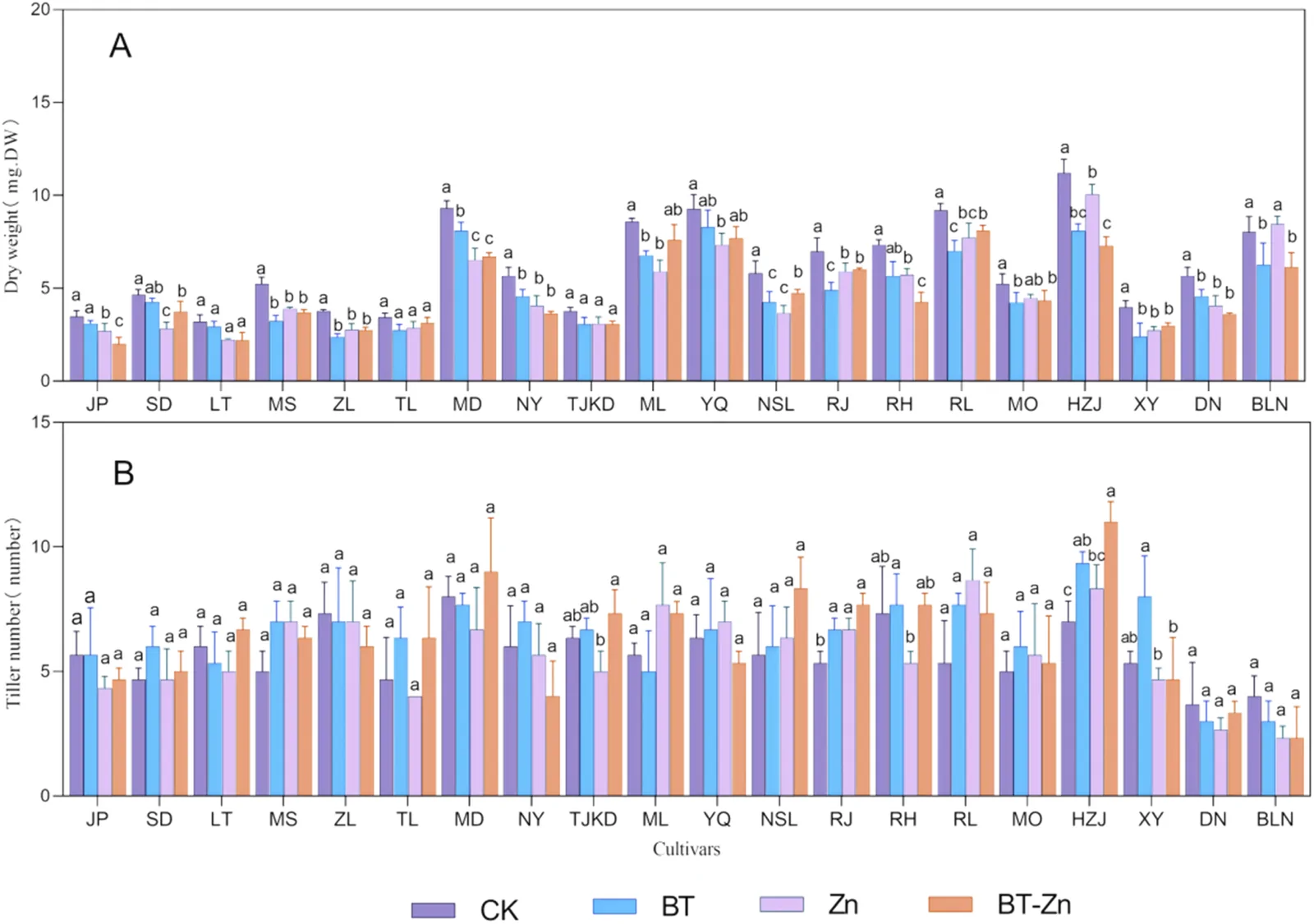
Effects of zinc and 2-mercaptothiazole stress on dry weight (A) and tillering number (B) of *Lolium perenne*.

### 3.3 Effects of combine Zn and 2-mercapto-benzothiazole stress on photosynthetic pigments of *L. perenne*

Fig. 3 illustrates that, alongside the trend of alterations in Ray hu during MBT stress, there was a considerable increase in chlorophyll *a*, chlorophyll *b*, carotenoids, and total chlorophyll compared to the control group. The increases were 105.98%, 117.89% (*P* < 0.05), 118.35%, and 108.19%, respectively. Other types exhibit a declining tendency in chlorophyll *a*, chlorophyll *b*, carotenoid, and total chlorophyll content. The concentrations of chlorophyll *a* ranged from 51.01% to 105.98%, 57.25% to 92.07%, and 59.77% to 97.32% for Zn and MBT individually and in conjunction, respectively. The variation in chlorophyll *b* content between treatment groups was 50.20–117.89%, 59.25–102.78%, and 50.36–112.56%, respectively (Fig. 3B). The variations in carotenoid content across all treatment groups were 52.16% to 118.35%, 46.59% to 93.75%, and 59.98% to 113.24%, respectively (Fig. 3C). The total chlorophyll shift rates ranged from 50.84% to 108.19%, 59.25% to 102.78%, and 50.36% to 113.56% (Fig. 3D).

**Fig. 3:**
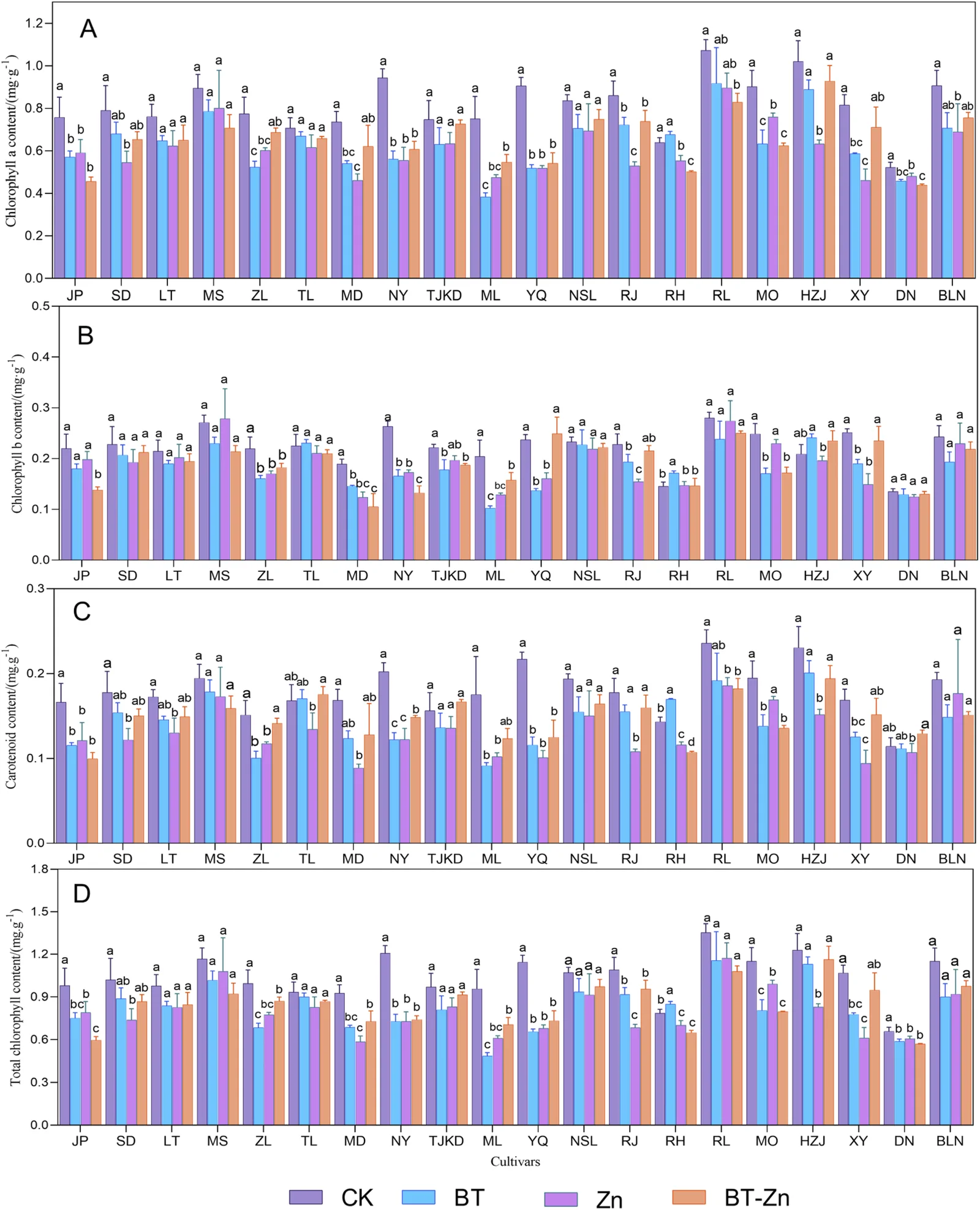
Effects of zinc and 2 mercaptothiazole-nickel stress on photosynthetic hormone content in *Lolium perenne*.

As can be seen from Fig. 4A, compared with the control group, under the combined stress of MBT treatments, Zn and MBT–Zn, the root activity of all treatment groups showed different degrees of decrease, and the decrease was significantly different. Fairy Green showed the smallest reduction of 64.63% compared to the control group treated with MBT. Royal Green had the largest reduction, 34.13 percent; Bomber had the largest reduction under Zn treatment, at 60.16 percent, and Rayleigh had the smallest reduction, at 95.23 percent, in the control group; Under MBT-Zn treatment, Turfgold decreased the most, 50.33% compared to control, and New Sealand decreased the least, 96.03% compared to control.

**Fig. 4:**
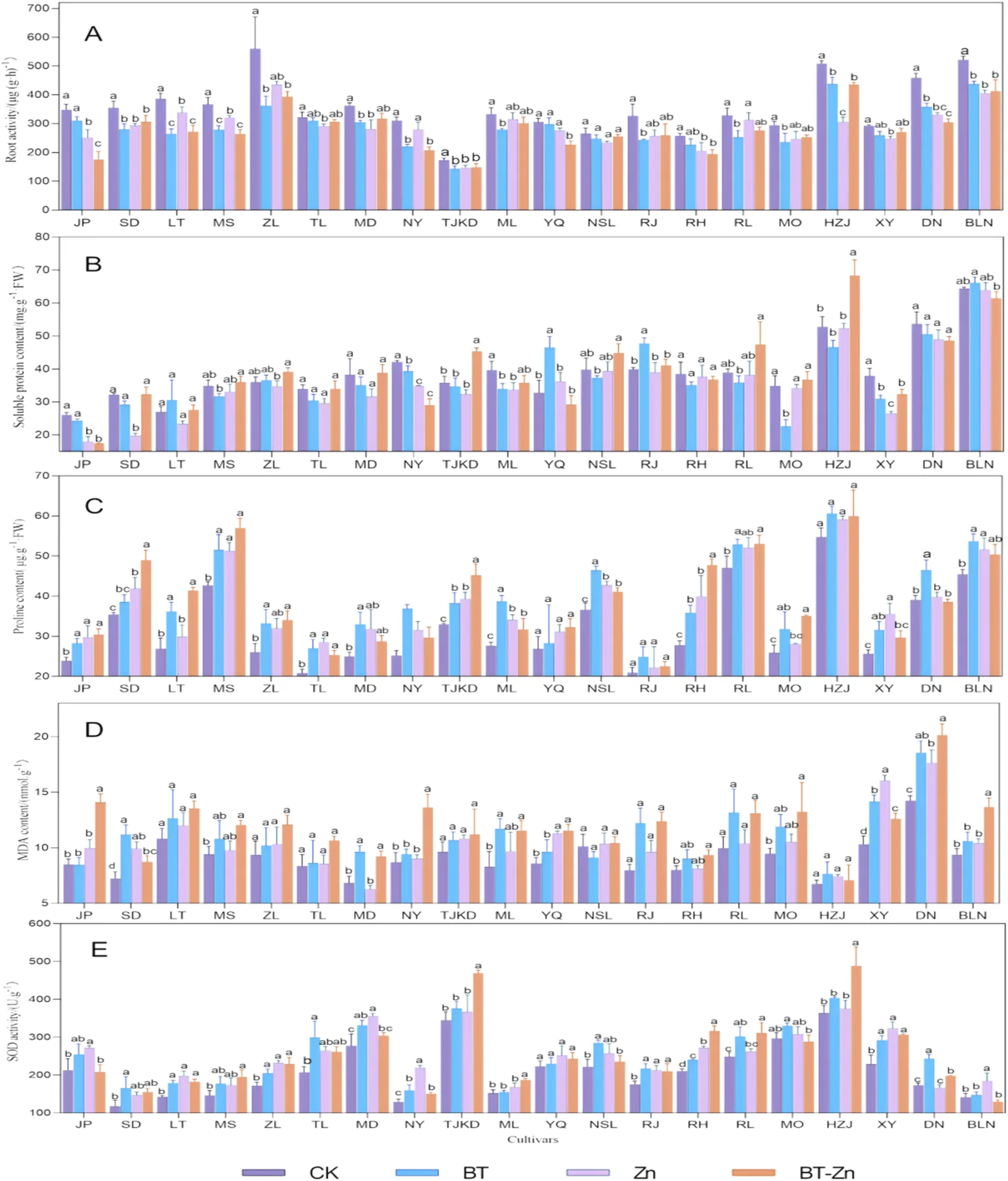
Effects of zinc and 2-mercaptothiazole stress on root activity (A), soluble protein content (B), proline (C), MDA content (D) and SOD activity (E) of *Lolium perenne*.

### 3.4 Effects of combine Zn and 2 mercapto-benzothiazole stress on physiological indexes of *L. perenne*

As shown in Fig. 4A, the root activity of each treatment group showed a downward trend compared with that of the control group. Under MBT treatment, the decrease of Yatsyn was smallest, which was 97.60% of the control group. Royal Green had the largest decrease, which was 64.63% of the control group; under Zn treatment, Ray Li showed the smallest increase, which was 95.23% of the control group. Ray hu Bomber had the greatest decrease, which was 60.16% of the control group; under MBT–Zn combined treatment, decrease of New Sealand was the smallest, which was 96.03% in the control group. Turfgold had the largest reduction, 50.33% of the control group.

Fig. 4B illustrates that the soluble protein levels of Turfsun, Royal Green, Mathilde, Ray hu, and Dina did not significantly differ from the control group when subjected to either individual or combined effects of Zn and MBT. Conversely, soluble protein levels of other varieties exhibited an increase, with Yatsyn demonstrating the most substantial enhancement under Zn and MBT stress. They were 142.18% and 110.63% of the control group, respectively. Bomber exhibited the most significant increase during MBT–Zn stress, recorded at 129.45% in the control group. Fig. 4C illustrates that, under both the individual and synergistic influences of Zn and MBT, proline accumulation levels in all treatment groups exceeded those of the control group, with a statistically significant difference. Under MBT treatment, the *L. perenne* with the smallest increase was Yatsyn, which was 105.47% of the control group. Nui increased the most, 146.39% in the control group. Under Zn treatment, Dina had the smallest increase, 101.88% of the control group; Ray hu showed the largest increase (143.64%) in the control group. Under MBT–Zn combined treatment, the decrease of New Sealand was the smallest, which was 98.86% of the control group. The increase of Ray hu was 171.83% of the control group.

MBT, Zn and MBT–Zn stress could increase the contents of MDA and antioxidants and the activity of SOD in *L. perenne* to a certain extent (Fig. 4D and E). Under MBT and Zn treatments, the MDA contents of New Sealand and Mathilde were decreased by 89.99% and 91.57%, respectively, with no significant difference, while other varieties were increased compared with control. Under MBT, Zn and MBT–Zn treatments, SOD activity changes of *L. perenne* were 101.79–144.54%, 96.39–114.50% and 91.41–150.05%, respectively. Combined exposure did not produce a uniform response across all cultivars and measured traits. Certain cultivars retained greater biomass, root length, or tillering under combined Zn and MBT exposure than under one or both individual treatments. In contrast, MDA accumulation, proline content, and SOD activity were frequently enhanced under combined exposure. These results indicate cultivar-dependent and trait-dependent interaction patterns but do not establish an antagonistic mechanism because contaminant uptake and internal accumulation were not determined.

Comparison of the three stress treatments showed that combined Zn and MBT exposure did not consistently produce the greatest response across all traits and cultivars. MDA accumulation, SOD activity, and proline content were frequently higher under combined exposure, indicating an enhanced oxidative and protective response. In contrast, the effects on biomass, plant height, root length, tillering, root activity, and photosynthetic pigments varied among cultivars. Several cultivars showed less inhibition of particular growth traits under combined exposure than under Zn or MBT treatment alone. These findings indicate that the interaction between Zn and MBT was nonadditive and depended on both the cultivar and the response variable.

### 3.5 Correlation analysis

Pearson correlation analysis was conducted on the tolerance coefficients of 15 indexes of *L. perenne* by SPSS (Table 3 and Fig. S1). The correlations among numerous indices attained highly significant levels. Notably, the concentrations of chlorophyll *a* and *b*, carotenoid content, total chlorophyll, and the relationship between carotenoids and chlorophyll *b* exhibited extremely significant positive correlations. Aboveground biomass had a positive correlation with dry weight and soluble protein content, while demonstrating a negative correlation with proline content. Underground biomass exhibited a positive correlation with plant height and a negative correlation with chlorophyll *a*, carotenoid content, and total chlorophyll content.

**Table 3:** Correlation coefficient matrix of tolerance coefficients of individual indexes^a^

Indicators	Above ground biomass	Subsurface biomass	Dry weight	Plant height	Root length	Tillering number	Chlorophyll *a*	Chlorophyll *b*	Carotenoids	Total chlorophyll	SOD	Proline content	MDA	Soluble protein	Root activity
Above ground biomass	1.000														
Subsurface biomass	−0.082	1.000													
Dry weight	0.634**	0.072	1.000												
Plant height	−0.055	0.349**	0.084	1.000											
Root length	0.068	0.049	0.072	−0.099	1.000										
Tillering number	0.232	0.139	0.008	0.281*	−0.117	1.000									
Chlorophyll *a*	0.165	0.296*	0.052	−0.133	−0.072	0.198	1.000								
Chlorophyll *b*	0.095	−0.153	0.112	−0.165	0.056	0.176	0.731**	1.000							
Carotenoid	0.161	0.292*	0.071	−0.127	−0.129	0.084	0.897**	0.658**	1.000						
Total chlorophyll	0.152	0.277*	0.067	−0.149	−0.042	0.205	0.988**	0.823**	885**	1.000					
SOD activity	−0.133	0.170	−0.115	0.003	−0.143	0.041	0.121	0.125	0.136	0.135	1.000				
Proline	0.328*	0.047	−0.208	0.012	−0.090	−0.160	−0.080	−0.107	−0.101	−0.089	0.375**	1.000			
MDA content	0.022	−0.206	−0.117	−0.179	−0.252	−0.076	−0.226	0.334**	−0.076	0.269*	−0.161	−0.079	1.000		
Soluble protein	0.393**	0.023	0.325*	−0.038	−0.112	0.288*	0.261*	0.178	0.198	0.249	−0.055	−0.101	−0.214	1.000	
Root activity	0.285*	0.031	0.253	0.347**	0.232	0.355**	0.197	0.117	0.015	0.197	0.100	−0.118	−359**	0.198	1.000

a * Stands for 0.01 < *P* < 0.05, ** stands for *P* < 0.01, * means 0.01 < *P* < 0.05, ** means *P* < 0.01.

Root activity was positively correlated with plant height and tillering number (*P* < 0.01), negatively correlated with aboveground biomass (*P* < 0.05), and negatively correlated with MDA content (*P* < 0.01). MDA content was significantly negatively correlated with chlorophyll *b* content (*P* < 0.01) and total chlorophyll content (*P* < 0.05). The above results showed that photosynthetic pigment, aboveground biomass, underground biomass, dry weight, root activity and MDA content could better reflect the tolerance of *L. perenne* seedlings under different stress treatments, and the effects of different indexes were different, so it was necessary to conduct comprehensive evaluation of these indexes.

### 3.6 Principal component and factor analysis

Table 4 presents eigenvalues, trait loadings, and contribution rates of principal components under different treatments. Under Zn stress, six principal components with eigenvalues greater than 1 were retained, explaining 84.78% of total variance, allowing reduction of 15 indicators into six comprehensive indices. The first principal component (CI1) was mainly associated with photosynthetic pigments, with high loadings for chlorophyll *b* (0.896), carotenoids (0.896), total chlorophyll (0.891), and chlorophyll *a* (0.864). The second component (CI2) represented biomass and morphological traits, including tillering number (0.749), aboveground biomass (0.723), dry weight (0.385), and root length (0.523). The third component (CI3) was related to root traits, particularly root activity (0.701) and root length (0.524).

**Table 4:** Principal component coefficients and contribution rates of comprehensive indexes of each treatment group

Indicators	Zn stress						MBT stress							Zn–MBT coercion			
	Cl_1_	Cl_2_	Cl_3_	Cl_4_	Cl_5_	Cl_6_	Cl_1_	Cl_2_	Cl_3_	Cl_4_	Cl_5_	Cl_6_	Cl_1_	Cl_2_	Cl_3_	Cl_4_	Cl_5_
Above ground biomass	0.276	0.723	−0.35	0.184	0.170	0.342	0.193	0.705	0.392	0.203	0.371	−0.057	0.474	0.586	0.252	−0.158	0.34
Subsurface biomass	−0.490	0.375	0.419	0.339	−0.435	−0.114	−0.555	−0.192	−0.022	0.532	0.064	0.476	0.199	−0.037	−0.186	0.832	−0.222
Dry weight	0.494	0.385	−0.491	0.450	0.264	0.237	−0.113	0.639	0.006	0.538	0.371	0.019	0.452	0.680	−0.166	0.067	0.228
Plant height	−0.435	0.008	0.293	−0.195	−0.271	0.652	−0.476	−0.465	−0.276	0.13	0.547	0.224	0.527	0.460	−0.241	0.061	−0.243
Root length	−0.028	0.523	0.524	−0.005	0.374	−0.302	0.181	0.499	−0.626	−0.056	0.016	−0.261	−0.465	0.238	0.415	0.47	0.350
Tillering number	−0.061	0.749	0.266	−0.390	−0.075	0.146	0.276	−0.485	0.320	−0.409	0.382	0.283	0.715	0.110	−0.441	0.005	−0.251
Chlorophyll *a*	0.864	−0.283	0.375	−0.100	−0.012	0.022	0.959	−0.079	−0.052	0.181	−0.004	0.115	0.866	−0.164	0.377	−0.179	−0.092
Chlorophyll *b*	0.896	−0.083	0.196	0.029	−0.059	0.083	0.916	−0.129	−0.165	0.115	−0.041	0.165	0.588	−0.208	0.115	0.515	0.279
Carotenoids	0.896	−0.278	0.083	0.018	−0.043	0.16	0.921	−0.146	−0.001	0.228	0.026	0.069	0.592	−0.229	0.649	0.008	−0.244
Total chlorophyll	0.891	−0.247	0.355	−0.078	−0.014	0.037	0.964	−0.089	−0.081	0.171	−0.012	0.121	0.891	−0.213	0.355	−0.035	−0.026
SOD activity	−0.535	−0.344	0.179	0.619	−0.02	0.142	0.535	−0.196	0.276	0.281	0.087	−0.326	0.49	−0.672	−0.201	0.141	−0.016
Proline	−0.364	−0.491	0.337	0.316	0.452	0.132	−0.362	−0.418	−0.313	0.54	0.042	−0.301	−0.062	−0.697	−0.328	−0.181	0.469
MDA content	−0.418	−0.188	−0.429	−0.547	0.266	0.040	−0.086	−0.065	0.847	0.033	0.182	−0.244	−0.794	0.284	0.177	−0.074	−0.252
Soluble protein	0.434	0.601	0.018	0.202	−0.09	−0.175	−0.131	0.607	0.241	0.096	−0.415	0.487	0.845	0.009	−0.109	−0.237	0.069
Root activity	−0.222	0.254	0.701	−0.129	0.38	0.159	0.213	0.442	−0.331	−0.433	0.544	0.095	0.751	0.407	−0.208	0.003	0.150
Characteristic roots	4.732	2.692	2.103	1.398	0.94	0.852	4.717	2.477	1.821	1.497	1.238	0.991	5.866	2.44	1.488	1.358	0.919
Contribution rate (%)	31.545	17.947	14.02	9.319	6.268	5.683	31.447	16.511	12.139	9.979	8.255	6.604	39.105	16.267	9.918	9.055	6.126
Above ground biomass	31.545	49.492	63.512	72.831	79.099	84.782	31.447	47.958	60.096	70.075	78.33	84.934	39.105	55.372	65.29	74.345	80.472

Under MBT stress, six principal components were also retained, accounting for 84.93% of total variance. CI1 was dominated by photosynthetic pigment traits, including total chlorophyll (0.964), chlorophyll *a* (0.959), chlorophyll *b* (0.916), and carotenoids (0.921). CI3 was primarily associated with physiological responses, particularly MDA content (0.847) and root length (−0.626). Under combined Zn MBT stress, five principal components with eigenvalues greater than 1 were retained, explaining 80.47% of total variance. The reduced number of components indicates stronger variable interactions under combined stress conditions. CI1 was mainly associated with photosynthetic and growth traits, including total chlorophyll (0.891), chlorophyll *a* (0.866), soluble protein (0.845), and tillering number (0.715). CI2 reflected stress-related physiological responses, including dry weight (0.680), SOD activity (−0.672), and proline (−0.697). CI3 was primarily associated with carotenoids (0.649).

### 3.7 Comprehensive evaluation of *L. perenne* varieties under combined Zn and 2-mercapto-benzothiazole stress treatments

The membership function value Mu *X* of each comprehensive index was calculated, and the *D* value was derived based on the corresponding weights to evaluate the tolerance of *L. perenne* varieties under different stress conditions (Table 5). The number of membership function indices varied among treatments according to the number of principal components retained. Six indices were used under Zn and MBT stress, while five indices were used under combined Zn MBT stress due to the lower number of principal components identified.

**Table 5:** Value of each comprehensive index (CI), index weight, *µ*(*X*), and comprehensive evaluation value (*D*)

Breed	Zn stress								MBT stress								Zn–MBT coercion						
	Mu (*X*_1_)	Mu (*X*_2_)	Mu (*X*_3_)	Mu (*X*_4_)	Mu (*X*_5_)	Mu (*X*_6_)	*D* Value	General evaluation	Mu (*X*_1_)	Mu (*X*_2_)	Mu (*X*_3_)	Mu (*X*_4_)	Mu (*X*_5_)	Mu (*X*_6_)	*D* Value	General evaluation	Mu (*X*_1_)	Mu (*X*_2_)	Mu (*X*_3_)	Mu (*X*_4_)	Mu (*X*_5_)	*D* Value	General evaluate
JP	0.610	0.174	0.445	0.525	0.635	0.357	0.466	14	0.512	0.572	0.184	0.363	0.751	0.476	0.480	13	0.000	0.591	0.547	0.284	0.454	0.254	20
SD	0.329	0.193	0.562	0.000	0.520	0.582	0.334	19	0.785	0.460	0.917	0.421	0.858	0.243	0.663	3	0.715	0.556	0.544	0.376	0.930	0.640	10
LT	0.610	0.238	0.678	0.513	0.016	0.700	0.494	11	0.516	0.357	0.492	1.000	0.460	0.782	0.554	9	0.668	0.300	0.500	0.532	0.672	0.558	14
MS	0.867	0.592	0.980	0.294	0.513	0.678	0.725	2	0.725	0.123	0.486	0.171	0.405	0.551	0.464	15	0.608	0.549	0.471	0.160	0.630	0.531	16
ZL	0.562	0.225	0.505	0.535	0.180	0.644	0.456	15	0.362	0.051	0.409	0.215	0.000	0.328	0.253	19	0.686	0.485	0.755	0.241	0.650	0.601	11
TL	0.793	0.295	0.724	0.690	1.000	0.687	0.673	4	0.951	0.312	0.431	0.371	1.000	0.391	0.646	4	0.892	0.826	0.718	0.691	0.648	0.816	2
MD	0.291	0.319	0.482	0.664	0.480	0.151	0.374	18	0.472	0.544	0.452	0.713	0.831	0.059	0.514	10	0.633	0.891	0.427	0.000	0.319	0.565	13
NY	0.029	0.537	0.842	1.106	0.166	0.816	0.452	16	0.144	0.130	0.377	1.285	0.743	0.504	0.395	16	0.148	0.746	0.561	0.731	0.325	0.399	19
TJKD	0.759	0.358	0.664	0.323	0.528	0.960	0.607	6	0.550	0.418	0.301	0.769	0.916	0.820	0.571	8	0.984	0.602	0.609	0.196	0.542	0.738	5
ML	0.143	0.738	0.756	0.102	0.587	0.853	0.446	17	0.000	0.265	0.500	0.644	0.864	0.219	0.300	20	0.602	0.868	0.000	0.461	0.281	0.541	17
YQ	0.254	0.967	0.518	0.414	0.882	0.000	0.496	10	0.244	1.333	0.565	0.116	0.673	1.001	0.587	7	0.400	0.964	0.385	1.461	1.085	0.684	8
NSL	0.690	0.481	1.000	0.241	0.388	0.192	0.592	8	0.770	0.488	0.000	0.160	0.574	0.151	0.467	14	0.962	0.956	0.444	0.609	0.552	0.826	1
RJ	0.397	0.872	0.311	0.544	0.362	0.342	0.493	12	0.677	0.528	1.563	0.608	0.515	0.566	0.742	1	0.768	0.964	0.625	0.828	0.369	0.766	3
RH	0.756	0.107	0.834	0.779	0.550	0.491	0.601	7	1.000	0.303	0.213	0.548	0.703	0.673	0.645	5	0.595	0.000	0.217	0.621	0.705	0.440	18
RL	0.905	1.276	1.355	0.330	0.780	0.732	0.974	1	0.825	0.484	1.051	0.421	0.701	0.523	0.708	2	0.773	0.847	0.289	0.833	0.520	0.716	7
MO	0.880	0.592	0.508	0.201	0.406	1.000	0.656	5	0.411	0.226	0.430	0.173	0.946	0.126	0.380	18	0.492	1.000	0.325	0.117	0.778	0.554	15
HZJ	0.669	0.784	0.000	0.479	0.000	0.843	0.524	9	0.762	0.120	0.399	0.120	0.605	0.999	0.513	11	1.000	0.566	0.459	0.548	0.420	0.751	4
XY	0.000	0.000	0.224	0.125	0.929	0.935	0.182	20	0.496	0.000	0.598	0.000	0.872	0.456	0.389	17	0.751	0.718	0.715	0.655	0.661	0.722	6
DN	1.000	0.114	0.280	0.035	0.212	0.091	0.468	13	0.840	0.514	0.609	0.538	0.422	0.000	0.602	6	0.569	0.520	0.960	0.684	0.000	0.577	12
BLN	0.961	0.510	0.104	0.904	0.932	0.707	0.698	3	0.593	0.750	0.369	0.205	0.331	0.181	0.489	12	0.476	0.914	1.000	0.292	0.922	0.642	9

The *D* value analysis revealed clear variation in tolerance among varieties and across treatments. Under Zn stress, *D* values ranged from 0.182 to 0.974. The variety XY showed the lowest tolerance, while RL exhibited the highest tolerance. Under MBT stress, *D* values ranged from 0.300 to 0.742, where ML showed the lowest tolerance and RJ showed the highest tolerance.

Under combined Zn MBT stress, *D* values ranged from 0.254 to 0.826. JP exhibited the lowest tolerance, whereas NSL showed the highest tolerance. Across all treatments, TL consistently demonstrated strong tolerance, ranking fourth under Zn stress, fourth under MBT stress, and second under combined stress. In contrast, ML consistently showed low tolerance, ranking seventeenth under Zn stress, twentieth under MBT stress, and seventeenth under combined stress. These results indicate that stress tolerance in *L. perenne* varies both among genotypes and across stress conditions, and that combined stress alters the weighting structure and relative performance of varieties.

## 4 Discussions

The individual and synergistic impacts of heavy metals and organic contaminants can adversely influence plant growth and many metabolic pathways, while plants modulate their internal environment to acclimate to stress *via* numerous physiological and biochemical mechanisms.^22,23^ The mechanism entails activation of antioxidant defense mechanisms to eliminate reactive oxygen species, manipulation of hormonal signaling pathways and modifications in nutrient absorption and distribution.^24^ Plants employ detoxification mechanisms, such as chelation and sequestration, to mitigate adverse effects of heavy metals. Moreover, stress-responsive gene expression and modifications in cell wall composition enable plants to cope with stress, hence promoting their survival and growth in adverse situations.^25^ In this study, it was found that under the single and combined action of Zn and MBT, there were differences in the dispersion degree of variation coefficient of 15 indicators and the change rate of the control group. In addition to tillering number, SOD activity, proline, and MDA content, the remaining indices had obvious inhibitory effects (Table 2), which can be ascribed to the intricate stress response mechanisms of the plants.

The noted disparities in the dispersion of variation coefficients and the alteration rates of the control group across the 15 indicators under both single and combined Zn–MBT stress illustrate intricate and diverse physiological responses of *Lolium perenne* to these stresses. The indices demonstrating distinct inhibitory effects, apart from tillering number, SOD activity, proline, and MDA concentration, likely indicate processes that are more susceptible to the oxidative and metabolic disturbances induced by Zn and MBT. Zinc poisoning can disrupt essential enzyme processes, including those related to photosynthesis and nutrient assimilation, whereas MBT may compromise membrane integrity and enzymatic activities. The fluctuations in SOD activity, proline accumulation, and MDA levels indicate that antioxidant mechanisms and osmotic regulation are engaged in alleviating stress, with SOD reducing ROS, proline serving as an osmoprotectant, and MDA levels representing lipid peroxidation resulting from oxidative stress. Nonetheless, other physiological processes, including growth and biomass accumulation, were more directly hindered by excessive metabolic burden imposed by combined contaminants, which surpassed plants' capacity to compensate or recuperate, resulting in diminished performance in these specific metrics. The inhibitory effects on most indices, excluding tillering number, SOD activity, proline, and MDA content, indicate that Zn and MBT stress interfered with normal physiological processes, including photosynthesis, nutrient absorption, and enzyme activity.^26^ The elevated SOD activity and proline levels may signify an antioxidant defense mechanism to alleviate oxidative stress, but MDA accumulation could imply lipid peroxidation. The disparate responses among indices may result from variations in stress sensitivity, plant adaptation strategies, and interactions between Zn and MBT.^27^

The coefficient of variation of underground biomass of *L. perenne* under Zn and combined Zn and 2-mercapto-benzothiazole stress treatments was the largest, while the coefficient of variation of aboveground biomass under MBT treatment was the largest, indicating that under Zn and MBT single and combined treatments, the coefficient of variation of biomass under MBT was the largest. Above-ground biomass and underground biomass of *L. perenne* responded more sensitively, and there were significant differences among different varieties of *L. perenne*. The elevated coefficient of variation in underground biomass under Zn and Zn–MBT combined treatment indicates that root development is especially vulnerable to Zn toxicity and MBT-induced stress. The heightened variability in aboveground biomass, along with MBT treatment, suggests that MBT primarily affects shoot growth. The notable disparities in biomass responses among varieties suggest genetic heterogeneity in stress tolerance, potentially due to variations in root architecture, antioxidant defense mechanisms, and the expression of stress-responsive genes. This variability can be utilized to create stress-resilient variants of *L. perenne*.

Growth responses varied substantially among cultivars. Biomass, plant height, root length, and dry weight generally declined following Zn and MBT exposure, although the magnitude of inhibition differed considerably among cultivars and some traits were maintained under combined exposure. This variability indicates that sensitivity to Zn and MBT cannot be generalized across *L. perenne* germplasm. The comparatively greater maintenance of particular growth traits in some cultivars under combined exposure also supports the finding that simultaneous Zn and MBT exposure was not uniformly more inhibitory than the individual treatments. This suggests that, in certain types, combined stress may trigger adaptive hormonal and physiological changes that temporarily mitigate tiller suppression, whereas sustained or escalated stress ultimately surpasses these defensive systems, leading to overall growth inhibition.^28^

Contrary to the initial expectation of uniformly greater toxicity, simultaneous Zn and MBT exposure did not affect every trait and cultivar in the same direction. Combined exposure intensified MDA accumulation and altered SOD activity in many cultivars, indicating a stronger oxidative challenge. However, several cultivars maintained greater biomass, root length, tillering, or pigment content under combined exposure than under one or both single stress treatments. Thus, the intensity of the oxidative response was not always directly associated with the degree of growth inhibition. Enhanced antioxidant and osmotic adjustment may have allowed some cultivars to maintain growth despite increased oxidative pressure. The higher proline, soluble protein, and SOD responses observed in several cultivars are consistent with this interpretation. The partial attenuation of certain growth effects may also indicate an antagonistic interaction between Zn and MBT at the exposure or uptake level. For example, interactions between the two compounds in the nutrient solution could potentially alter Zn availability, MBT stability, or their uptake by roots. However, Zn speciation, MBT degradation, and tissue concentrations of both contaminants were not measured in this experiment. These mechanisms therefore remain possible explanations rather than demonstrated processes. Overall, the results support a trait-dependent and cultivar-dependent interaction in which combined exposure intensified some oxidative responses but did not uniformly intensify growth inhibition.

Photosynthetic pigment responses also differed among cultivars. Most cultivars showed reductions in chlorophyll and carotenoid contents under contaminant exposure, consistent with previous reports of pigment sensitivity to excess Zn and other environmental contaminants. In contrast, Ray hu maintained or increased several pigment traits under MBT exposure, indicating a cultivar-specific response. Photosynthesis is the basis of plant survival, and photosynthetic parameters can reflect the intensity of photosynthesis and indirectly reflect the growth of plants.^29,54^ Changes in chlorophyll content represent the photosynthetic capacity of the whole plant,^30^ and carotenoids are non-enzymatic antioxidants. They can promote pigment protection and lipid peroxidation under external stress.^31,56^ The results of this study showed that the change in chlorophyll *a* content was related to the variety and stress conditions. Under MBT stress, the total amount of chlorophyll *a*, chlorophyll *b*, carotenoids, and chlorophyll in Ray hu increased, indicating that MBT had a promoting effect on the synthesis and production of chlorophyll in Ray hu. The increase in chlorophyll *a*, chlorophyll *b*, carotenoids, and total chlorophyll in Ray hu under MBT exposure indicates a cultivar specific physiological response that differed from the general decline observed in most other cultivars. This response may reflect greater pigment stability or more effective physiological adjustment to MBT exposure. However, the underlying mechanism was not directly investigated in the present study. This promotional impact may be linked to increased activation of chlorophyll production enzymes, greater stabilization of photosynthetic pigments, or decreased pigment degradation owing to more effective antioxidant defense in Ray hu. MBT may also influence stress-related signaling and hormonal pathways, resulting in activation of photosynthesis-related genes and enhanced carotenoid production, which protects chlorophyll from oxidative damage by scavenging excess reactive oxygen species. Collectively, these processes suggest that Ray hu exhibits superior physiological adaptability and detoxifying ability, enabling it to sustain or even augment photosynthetic pigment production under MBT stress, unlike more sensitive types. This may be due to the activation of stress-induced signaling pathways that enhance the expression of genes related to chlorophyll synthesis.^32,55^ Furthermore, MBT may enhance the production of antioxidants, such as carotenoids, which protect chlorophyll from oxidative damage, hence augmenting photosynthesis and promoting plant development.^33^ This cultivar-specific response highlights the substantial variation in physiological sensitivity to MBT among *L. perenne* cultivars. This, wherein low stress levels elicit favorable physiological responses.^34^ The content of four photosynthetic pigments of the remaining varieties showed a decreasing trend under the three treatments; especially, Zn treatment had a great effect on the photosynthetic pigments of all varieties. The single and complex effects of MBT and Zn reduced the content of photosynthetic pigments and inhibited the photosynthetic ability of *L. perenne* leaves. The reduction of photosynthetic pigments under Zn and MBT stress is likely due to oxidative damage to chloroplasts and the disruption of pigment production processes.^35^ Zn poisoning can substitute magnesium in chlorophyll molecules, resulting in pigment breakdown, whereas MBT may disrupt enzyme function and gene expression related to pigment synthesis.^36^ The cumulative stress may intensify these effects, resulting in more significant decreases in photosynthetic pigments. Decreased pigment concentration hinders light absorption and electron transfer, hence obstructing photosynthesis and restricting plant development. Variations in antioxidant defense systems and stress tolerance mechanisms among varieties may affect the extent of these effects.^37^ This is similar to the conclusion that Zn stress has the strongest inhibitory effect on photosynthetic pigments of *L. perenne* under single or combined pollution of lead (Pb), zinc (Zn), and cadmium (Cd).^38^

Higher MDA accumulation together with changes in SOD activity and proline content under Zn and MBT exposure indicates an altered oxidative and protective physiological state in *L. perenne*. MDA accumulation is consistent with increased lipid peroxidation, whereas changes in SOD activity indicate modification of one enzymatic component involved in superoxide metabolism. Increased proline may contribute to osmotic adjustment and cellular protection. However, these responses were cultivar-dependent and were not uniform across treatments. Because individual reactive oxygen species, hydrogen peroxide, CAT, POD, APX, glutathione, and membrane electrolyte leakage were not measured, the present results cannot determine the overall antioxidant capacity or establish whether reactive oxygen species production exceeded detoxification capacity. MDA, a byproduct of lipid peroxidation (LPO), is often evaluated as a biomarker for the early stages of oxidative stress response.^39^ The level of LPO in plants can be represented by MDA content in leaves.^40^ The SOD system is the first line of defense for the body to resist oxidative stress, and superoxide dismutase (SOD) can transform superoxide free radicals into less toxic H_2_O_2_.^41^ The changes of MDA content and SOD activity showed that the combined treatment of MBT and Zn induced more severe oxidative stress than that of MBT and Zn alone (Fig. 4D and E), likely attributable to the production of reactive oxygen species (ROS) from both stressors, the elevated MDA levels and modified SOD activity under concurrent MBT and Zn treatment suggest that simultaneous exposure induces greater oxidative stress than either agent individually, mainly due to additive or synergistic overproduction of ROS from both pollutants. Zinc poisoning can impair electron transport chains and redox equilibrium, whereas MBT disrupts cellular metabolism and membrane-related functions, thereby increasing ROS production beyond the detoxifying capabilities of the antioxidant system. MDA content and SOD activity varied substantially among cultivars and exposure treatments. Because MDA is commonly used as an indicator of lipid peroxidation, its increase in several cultivars is consistent with greater oxidative damage to cellular lipids. Changes in SOD activity indicate that the antioxidant response was modified under Zn and MBT exposure. Higher SOD activity in some cultivars may represent an enhanced capacity to convert superoxide radicals, whereas limited or reduced activity may indicate a weaker response to oxidative pressure. However, individual ROS species, hydrogen peroxide content, and membrane permeability. Under combined stress, the antioxidant system becomes partially overwhelmed, resulting in inadequate ROS scavenging and exacerbated oxidative damage, which accounts for the heightened oxidative stress reported with MBT–Zn co-exposure. Zn can facilitate the generation of ROS *via* Fenton-like processes, whereas MBT may interfere with electron transport chains, hence exacerbating ROS levels.^42^ The cumulative stress may surpass the plant's antioxidant defense mechanism, resulting in heightened lipid peroxidation (as evidenced by MDA) and modified SOD activity.^43^ The synergistic impact may arise from the interaction between Zn and MBT, amplifying their respective toxicities and leading to increased oxidative damage to cellular components.^44^ The combined effects of Zn and MBT stress likely stem from their interactive disruption of cellular redox balance and detoxification mechanisms, with Zn interfering with metal homeostasis and enzyme function, while MBT modifies membrane integrity and metabolic processes, together increasing the production of ROS. Excess zinc can disrupt mitochondrial and chloroplast electron transport, leading to heightened superoxide generation, whereas MBT may inhibit critical enzymes and induce membrane instability, thereby allowing the diffusion and buildup of ROS. The combined impacts surpass antioxidant defenses, hasten lipid peroxidation, protein oxidation, and membrane damage, ultimately exacerbating oxidative harm to cellular components beyond the impact of each stressor alone.

Proline (Pro) and soluble protein (SP) are the main osmoregulators of plant cells.^45^ They also play an important role in the stability of biomacromolecular structure, membrane, and subcellular structure, and the removal of reactive oxygen species.^46,52^ In this study, it was found that under the single or combined action of Zn and MBT, the root activity of all *L. perenne* varieties decreased, the level of proline accumulation increased significantly, and the soluble protein content of some varieties with strong tolerance increased. Combined with the results of correlation analysis among various indexes (Table 3), the reduction in root activity under Zn and MBT stress is likely due to oxidative damage and disruption of cellular processes, whereas the notable increase in proline accumulation indicates a stress tolerance mechanism, with proline functioning as an osmoprotectant and antioxidant.^47,53^ The reduction in root activity of *Lolium perenne* varieties under single or combined Zn and MBT stress is due to toxicity from metal and organic contaminants, which compromise membrane integrity, enzyme functionality, and cellular respiration in root tissues, thus hindering water and nutrient absorption. The substantial buildup of proline serves as a recognized adaptive response to stress, acting as an osmoprotectant, reactive oxygen species scavenger, and stabilizer of proteins and membranes, aiding in the maintenance of cellular homeostasis under Zn–MBT-induced oxidative and osmotic stress. The elevated soluble protein content in more tolerant varieties likely indicates the augmented production of stress-responsive proteins, such as antioxidant enzymes, metal-chelating proteins, and molecular chaperones, which together facilitate detoxification, redox equilibrium, and cellular protection. The observed physiological adaptations indicate that differences in tolerance among varieties are mainly determined by the efficacy of osmotic control, antioxidant defense and protein-based protective mechanisms that alleviate Zn–MBT-induced damage. The elevation of soluble protein concentration in tolerant cultivars may signify the production of stress-related proteins, like heat shock proteins or antioxidant enzymes, which assist in alleviating stress-induced damage.^48,54^ Correlation analysis (Fig. S1) likely identified associations between stress-responsive characteristics and plant tolerance, indicating that tolerant cultivars utilize a mix of antioxidant defense, osmotic adjustment, and protein-based protective mechanisms to withstand Zn and MBT stress.^49,50^ This study showed that under hydroponic conditions, *L. perenne* maintains the balance of free radical metabolism and regulates the content of soluble protein by enhancing the activity of antioxidant enzymes. Osmotic substances regulate the permeability and accumulate higher proline content to enhance tolerance. Plant stress resistance is usually a quantitative trait controlled by multiple genes. Plant traits of different genotypes have different responses to external stress, and there are obvious interaction effects among multiple traits.^51^

The assessment of oxidative responses in this study was limited to SOD activity, MDA content, proline accumulation, and soluble protein content. MDA provided evidence of lipid peroxidation, while changes in SOD activity indicated modification of the enzymatic response responsible for converting superoxide radicals. Proline accumulation provided additional information on osmotic adjustment and cellular protection but did not directly quantify reactive oxygen species or antioxidant capacity. Because CAT, POD, APX, glutathione, hydrogen peroxide, and electrolyte leakage were not measured, the present study cannot characterize the complete antioxidant network or determine whether hydrogen peroxide generated after SOD activity was effectively removed. It also cannot distinguish between increased reactive oxygen species production, insufficient detoxification, and direct membrane injury as the main cause of MDA accumulation. Therefore, increased SOD activity should not be interpreted as evidence that the entire antioxidant defence system was enhanced. Likewise, changes in MDA and proline support an altered oxidative and protective state but do not establish the magnitude or precise biochemical origin of oxidative stress. Future studies should integrate enzymatic antioxidants, nonenzymatic redox metabolites, reactive oxygen species measurements, and membrane integrity indicators to clarify the coordinated response of *L. perenne* to Zn and MBT.

The ability to maintain biomass, photosynthetic pigments, root activity, and antioxidant regulation under contaminant exposure is relevant to the initial selection of stress-tolerant plant material. However, physiological tolerance should not be equated with phytoremediation efficiency. Plants may tolerate Zn through root exclusion, cell wall retention, vacuolar sequestration, or restricted shoot translocation, and these processes may result in either high or low tissue accumulation. Similarly, MBT may be absorbed, retained at the root surface, translocated, degraded, or transformed into metabolites. Because Zn and MBT concentrations were not measured in roots, shoots, or nutrient solutions, the present study cannot determine which of these processes occurred. Accordingly, the identified cultivars should be regarded as physiologically tolerant candidates rather than confirmed phytoremediation. Confirmation of remediation performance would require measurement of Zn accumulation in roots and shoots, quantification of MBT and its transformation products, analysis of residual contaminants in the exposure medium, and calculation of contaminant-specific bioconcentration, translocation, and removal factors. The absence of these measurements limits conclusions concerning contaminant extraction but does not affect the comparative physiological ranking of cultivars, which was the principal purpose of this study.

The hydroponic system used in this study allowed precise control of Zn and MBT concentrations and supported consistent comparisons among *L. perenne* cultivars. However, this system cannot reproduce the physical, chemical, and biological complexity of natural soils. The use of sterilized nutrient solutions minimized microbial activity, thereby excluding microbial processes that may alter contaminant toxicity. Soil microorganisms can influence Zn speciation through changes in rhizosphere pH, redox conditions, chelation, and immobilization, while microbial communities may degrade or transform MBT into products with different mobility, persistence, and toxicity. The absence of soil organic matter and mineral particles also eliminated adsorption, complexation, precipitation, and sequestration processes that regulate the freely available fractions of Zn and MBT. In soil, these processes may reduce direct root exposure, although changes in pH, dissolved organic matter, or microbial activity may also remobilize retained contaminants and increase their availability. Moreover, hydroponic solutions expose roots to relatively uniform contaminant concentrations, whereas natural soils contain spatial and temporal gradients in contaminant distribution, moisture, aeration, nutrient availability, and root contact. The toxicity observed under hydroponic conditions may therefore be greater, lower, or qualitatively different from that occurring in contaminated soils. Accordingly, the present results represent comparative physiological responses under controlled exposure conditions rather than direct predictions of plant performance in natural environments. Soil-based pot experiments and field trials are required to verify the tolerance rankings and determine how soil properties and rhizosphere processes modify the individual and combined effects of Zn and MBT.

This investigation revealed significant disparities in growth, development, physiological and biochemical features among various *L. perenne* types. This signified that each genotype has developed its own defense mechanism to manage stress. The tolerance of *L. perenne* varieties under individual and combination MBT and Zn stress could not be adequately assessed using only a limited number of indices. The observed variations in growth, development, and physiological responses across *L. perenne* types under MBT and Zn stress suggest that each genotype has developed unique adaptation mechanisms to cope with stress. The complex nature of stress tolerance mechanisms, involving several biochemical pathways and physiological processes, complicates the evaluation of tolerance using a restricted set of markers. Diverse species may employ different strategies, such as antioxidant defense, osmotic adjustment, or hormonal modulation, to mitigate adverse impacts of stress. An exhaustive evaluation of stress tolerance requires a comprehensive approach, incorporating many indices and considering interrelations among different physiological and biochemical systems. In this study, the tolerance coefficient of a single index was employed to assess the tolerance of varieties, thereby mitigating inherent differences among them. Comprehensive evaluations derived from membership function values, principal component analysis, and cluster analysis was utilized to rank the varieties. Three high-tolerance types and one high-salt-sensitive variety were chosen under individual and combined MBT and Zn stress conditions, respectively.

## 5 Conclusion

This study demonstrated substantial cultivar dependent and trait dependent variation among 20 *Lolium perenne* cultivars exposed to Zn, MBT, and their combination under controlled hydroponic conditions. Zn and MBT exposure generally reduced biomass accumulation, plant height, root growth, root activity, and photosynthetic pigment content, while changes in proline, MDA, soluble protein, and SOD activity indicated contrasting physiological responses among cultivars. Combined exposure did not consistently produce greater effects than either individual contaminant, indicating that responses depended on both cultivar and measured trait. The resulting physiological rankings identify contrasting cultivars that may be useful for further investigation, but physiological tolerance should not be interpreted as evidence of phytoremediation capacity. Zn and MBT uptake, accumulation, translocation, transformation, and removal were not measured, and the mechanisms underlying the observed cultivar differences therefore remain unresolved. Further studies incorporating contaminant uptake measurements, longer exposure periods, soil based experiments, and field validation are required to determine the environmental relevance and practical performance of the identified cultivars.

## Conflicts of interest

The authors declare no conflicts of interest.

## Supplementary Material

RA-016-D6RA04307K-s001

## Data Availability

All the data generated for this study are available in the tables and figures of the manuscript. Supplementary information (SI) is available. See DOI: https://doi.org/10.1039/d6ra04307k.
