# Supplementary material for: Effects of combined zinc and 2 mercaptobenzothiazole exposure on growth and physiological characteristics of Lolium perenne cultivars
Source: RSC Adv. 2026 Sep 24;16(45):57156–75. doi: 10.1039/d6ra04307k (PMC13602301; doi:10.1039/d6ra04307k)
Supplement: Supplementary file 1 [file RA-016-D6RA04307K-s001.pdf]

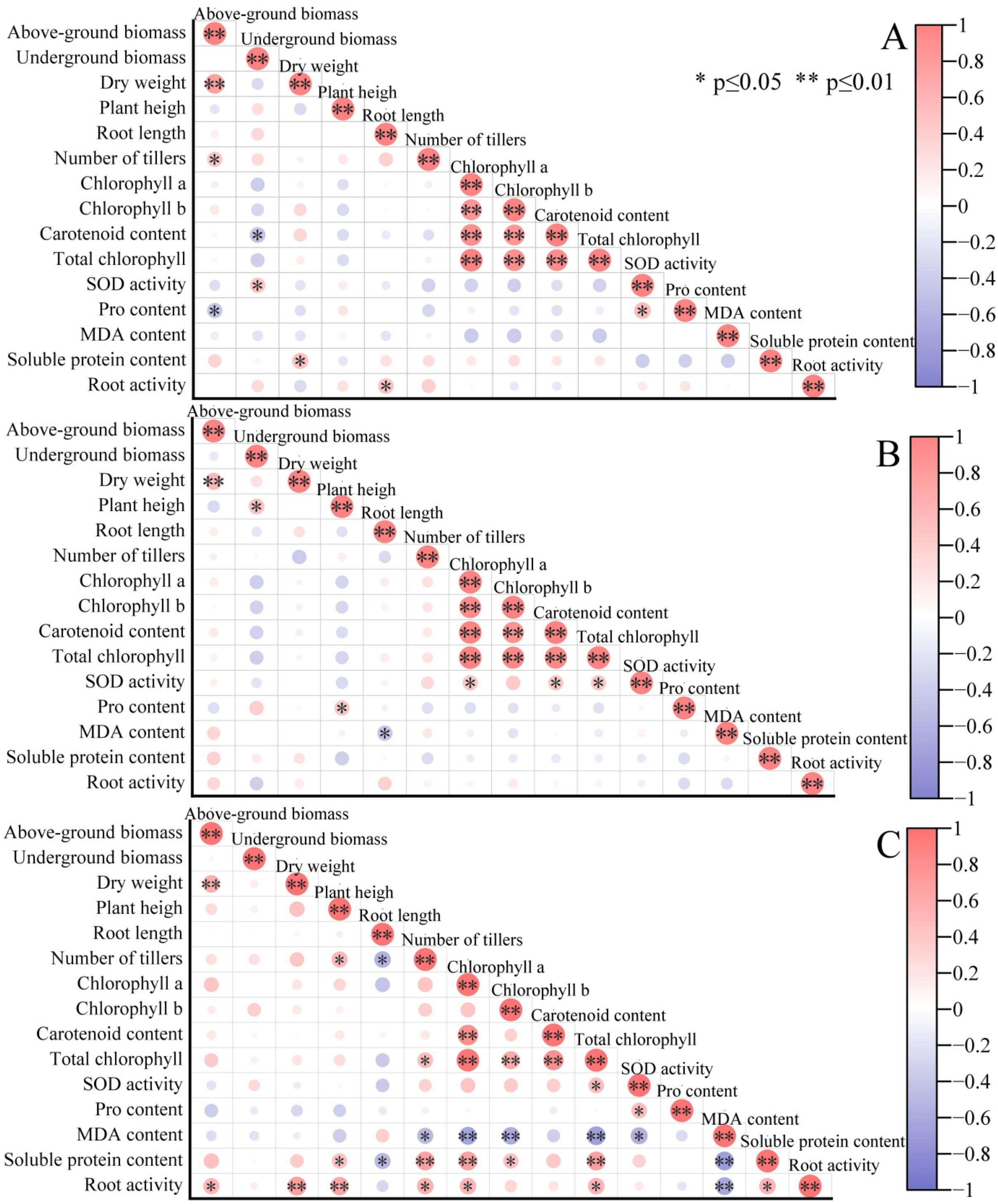

Figure S1 correlation analysis effects of zinc and 2 mercaptothiazole-nickel stress on photosynthetic hormone content in Lolium perenne
